# Above knee amputation following periprosthetic joint infection after total knee arthroplasty: a fatal outcome

**DOI:** 10.1093/jscr/rjae806

**Published:** 2024-12-21

**Authors:** Catherine Purcell, Rajkumar Gangadharan, George Ampat

**Affiliations:** School of Medicine, University of Liverpool, Cedar House, Ashton Street, L69 3GE, United Kingdom; Trauma and Orthopaedics, Aintree University Hospital, Lower Ln, Fazakerley, Liverpool L9 7AL, United Kingdom; School of Medicine, University of Liverpool, Cedar House, Ashton Street, L69 3GE, United Kingdom

**Keywords:** arthroplasty, replacement, knee, infection, amputation, outcomes

## Abstract

Primary total knee arthroplasty (TKA) is a successful and cost-effective procedure for which demand is increasing annually. Outcomes are generally good with satisfaction rates of 70%, so the procedure is commonly used in osteoarthritis management to improve mobility and alleviate pain. Above knee amputation (AKA) is a devastating complication of TKA. AKA is associated with high mortality, phantom pain, and non-ambulatory disability. In the US, the incidence of AKAs due to periprosthetic joint infection (PJI) is rising drastically, with the proportion of AKAs performed due to PJI having almost quadrupled from 1998 to 2013. We present the case of a patient who developed severe uncontrolled infection following routine TKA, resulting in an above-knee amputation and ultimately death. Due to the extremity of the outcome and the rising incidence of the complication involved, this is an important case to discuss.

## Introduction

Primary total knee arthroplasty (TKA) is very successful and cost-effective, consequently it is used routinely for the treatment of debilitating osteoarthritis [1]. Currently in the UK, over 90 000 TKA procedures are carried out annually [2]. TKA is typically associated with good outcomes by increasing knee function and providing pain relief [1]. However, studies suggest that following this procedure up to 14.4% of patients experience a major complication which may include surgical site infection, reoperation, or mortality [3].

A more frequently emerging devastating complication of TKA is above-knee amputation (AKA). This complication is associated with high mortality, phantom pain, reduced mobility and the need for further operations after stump closure [4]. AKA following a TKA is typically regarded as rare, with a prevalence ranging between 140 and 410 AKAs per 100 000 TKAs [6]. This approximately equates to 1 in 400 arthroplasties. A more recent systematic review has estimated the prevalence could be as high as 10% [5], marking a significant increase in comparison to previous estimates.

In the United States, the incidence of AKAs due to periprosthetic joint infection (PJI) is rising drastically [6]. In 1998 1% of all AKAs were due to PJI. Comparatively, in 2013 4% of all AKAs occurred secondary to infection after arthroplasty [6]. Within this 15-year period AKA incidence due to PJI has quadrupled, highlighting the need for increased awareness amongst both practitioners and patients.

## Case report

**Figure 1:**
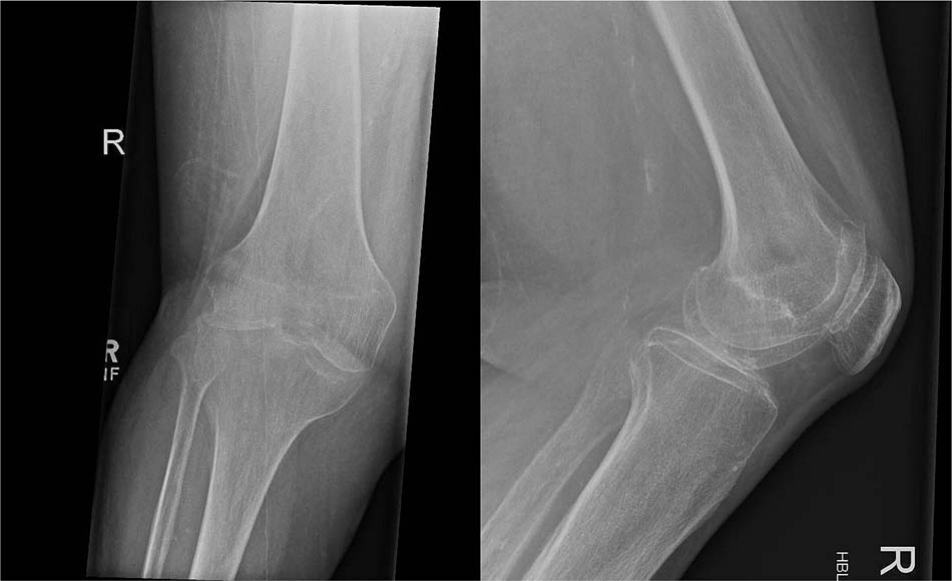
Pre-operative AP and lateral X-rays of the knee.

**Figure 2:**
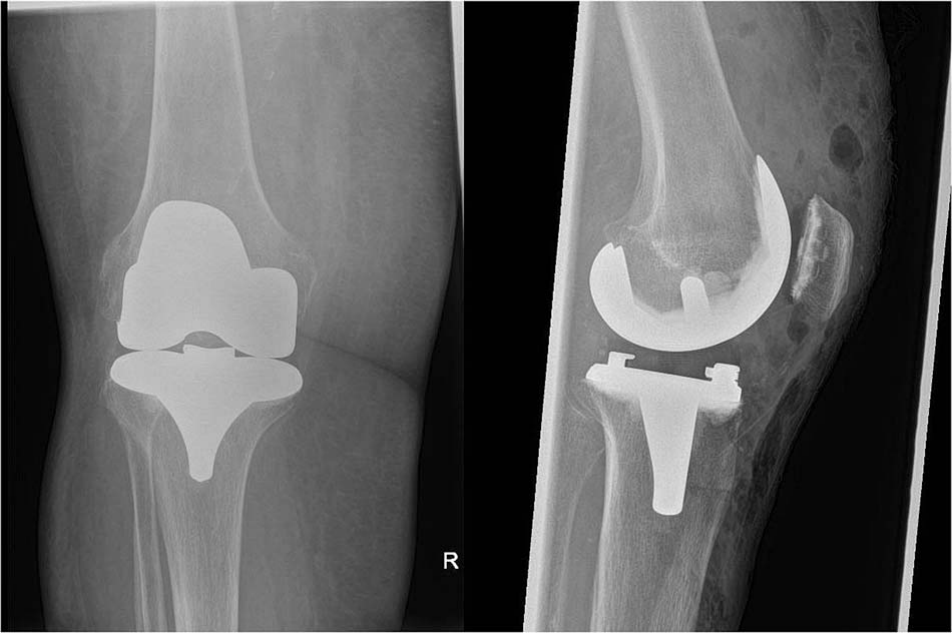
Post-operative AP and lateral X-rays of the knee.

In July 2023, a 75-year-old female patient underwent elective right total knee replacement with patellar resurfacing for osteoarthritis (Fig. 1). Prior to this procedure she was able to mobilize using a walker. Her comorbidities included hypertension, type 2 diabetes mellitus, diverticulosis, Parkinson’s disease, and a body mass index of 33.2. Surgery was uneventful so she was discharged home and requested to continue with rehabilitation (Fig. 2). Two weeks postoperatively she noticed redness and warmth surrounding the knee joint and a further 2 weeks later she felt a “pop” whilst walking. She then presented with a painful, swollen right knee on which she was unable to weight bear. X-rays identified a closed right patellar sleeve fracture. It was assumed that the pain and inflammation was due to the fracture and not due to infection and the decision was taken to treat the fracture non-operatively. The knee was splinted, and she was allowed to touch weight bear with a frame.

**Figure 3:**
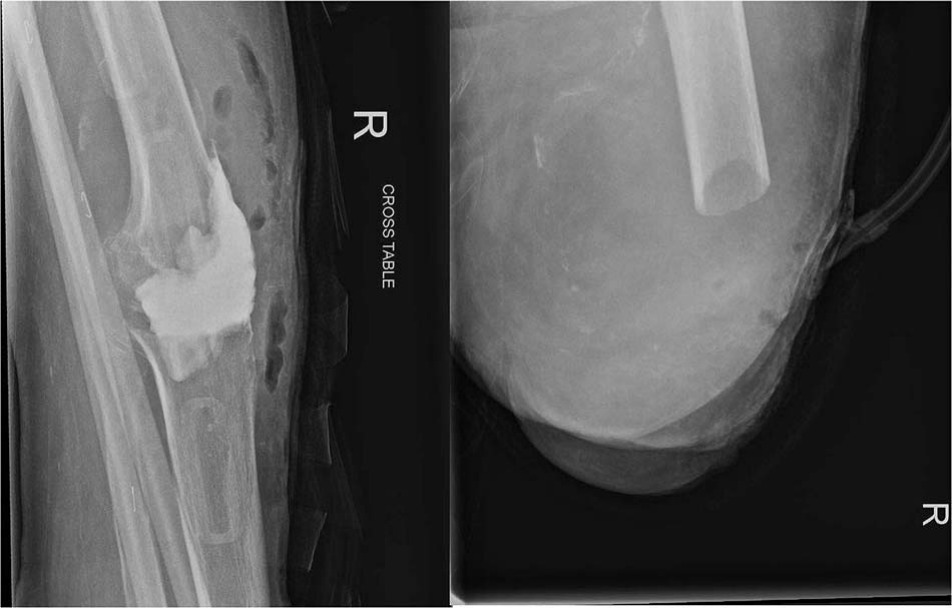
On the left X-ray of the knee after removal of prosthesis and insertion of antibiotic-impregnated cement spacer and on the right X-ray of the stump following amputation.

Over the following month, the skin above the fractured patella became necrotic, resulting in an open discharging wound. It was now clear that infection was the issue. With Orthoplastics involvement, she underwent multiple tissue samplings, patellectomy, removal of implants, and insertion of an antibiotic impregnated cement spacer (Fig. 3). Over the ensuing days the infection was uncontrollable despite further debridement and appropriate multi-drug antibiotic therapy. Following a multi-disciplinary team decision, a staged Above Knee Amputation (AKA) was performed (Fig. 3) with negative pressure dressings applied in the interim. Patient was discharged 4 weeks later on achieving closure of the stump. Mobility at discharge was limited to hoist transfers. Unfortunately, the patient passed away 8 weeks later.

## Discussion

TKA is one of the most effective and successful orthopaedic surgeries for debilitating osteoarthritis [1], with patient satisfaction rates as high as 70% [7]. Of the complications following arthroplasty, infection resulting in AKA is becoming increasingly prevalent. Between 1998 and 2013 there was a 3.6% rise in the number of AKAs performed due to PJI [6].

The outcomes of patients who have needed an AKA secondary to PJI are typically poor. One study over a 13-year period showed patients who underwent an AKA due to PJI had a 50% 5-year mortality rate and the remaining 50% experienced a reduction in mobility and independence [8]. Patients who require AKA following TKA often have a long and complex journey, requiring on average 5.5 procedures in the period between initial arthroplasty and final amputation [9]. There is evidence to suggest socioeconomic disparities exist within this cohort, with poorer patients at greater risk of needing amputation following PJI [10].

The case we present supports the current literature and demonstrates the reality of amputation as a devastating complication of knee arthroplasty. Due to uncontrolled PJI our patient, who prior to intervention was able to mobilize using a frame, ultimately became completely immobile requiring hoist transfers just 6 months post operatively. Within a further 2 months she sadly passed away. Her comorbidities not only increase her risk of PJI [11], but would also compromise her ability to recover from an AKA. This emphasizes the importance of careful patient selection and risk stratification prior to elective arthroplasty to try and avoid life threatening complications.

Due to diagnostic confusion at the start of this patients’ post-operative period, the PJI was initially missed. The patellar fracture sustained at 4 weeks masked the underlying infection that was occurring simultaneously, which led to delayed treatment. Despite step-wise surgical escalation and management from the multidisciplinary team once PJI was confirmed, the outcome was still poor, underlying early diagnosis as crucial in the management of infection.

This case highlights the need for increased awareness of amputation as a potential serious complication of routine knee arthroplasty, especially due to its rising incidence. Whilst TKA significantly improves quality of life for most patients, those with multiple PJI risk factors require explicit discussion of the increased potential for complications. Pre-operative modifiable risk factors should be optimized prior to surgery, in the case of this patient that would include weight loss and optimal diabetic control. Combining this with further post-operative monitoring for high-risk patients may help to reduce the rising incidence of this devastating complication.
